# Mediastinal mass as an unusual presentation of paragonimiasis in a child: a case report and review of diagnostic challenges

**DOI:** 10.1186/s12879-026-12893-8

**Published:** 2026-02-16

**Authors:** Shuchun Yang, Li Song, Qingwei Guo

**Affiliations:** 1 Department of Pediatric Intensive Care, Jinan Children’s Hospital, Jinan, Shandong China; 2 Department of Hematology and Oncology, Jinan Children’s Hospital, Jinan, Shandong China

**Keywords:** Child, Diagnostic dilemma, Mediastinal mass, Neglected diseases, Paragonimiasis

## Abstract

**Background:**

Paragonimiasis is a parasitic disease characterized by diverse clinical manifestations, among which pulmonary involvement is the most frequently observed. However, there are no documented cases of paragonimiasis presenting as a mediastinal mass. We report a case of paragonimiasis associated with such a presentation.

**Case presentation:**

A large mediastinal mass was identified in a 13-year-old child. Due to the limited efficacy of antibiotics and indeterminate diagnosis, thoracoscopic surgery was performed. Histopathological findings were consistent with paragonimiasis. The patient responded well to praziquantel treatment.

**Conclusions:**

This report reveals a novel, mediastinal presentation of paragonimiasis, a neglected disease, which posed a significant diagnostic dilemma due to its atypical features. Diagnostic thoracoscopy was essential for obtaining a definitive diagnosis, highlighting the importance of considering invasive diagnostic procedures when conventional approaches fail. The present case serves to remind clinicians to include paragonimiasis in the differential diagnosis of a mediastinal mass with chronic cough, especially in endemic areas or with a relevant dietary history.

Paragonimiasis is a zoonotic disease caused by food-borne trematodes of the genus *Paragonimus*. It affects approximately 23 million people worldwide, primarily in Asia [1, 2]. In China, pediatric data on its symptomatology, diagnosis, and management are limited according to the Chinese Center for Disease Control [3]. Paragonimiasis can present with a variety of nonspecific symptoms, rendering diagnosis challenging. Although pulmonary involvement is most common, ectopic migration of larvae can lead to infection in diverse sites such as the brain, liver, and subcutaneous tissues [4–9]. However, presentation as a mediastinal mass has not been previously documented, to the best of our knowledge. The present report describes such a case.

## Case presentation

A 13-year-old female patient from Heze county (Shandong, China), presented to our hospital with a 2-month history of recurrent episodes of cough and occasional brownish expectoration. The symptoms had persisted over this period without notable progression. The patient reported no fever, tachypnea, night sweats, weight loss, chest or abdominal pain, or diarrhea. Physical examination upon admission revealed no abnormalities.

**Fig. 1:**
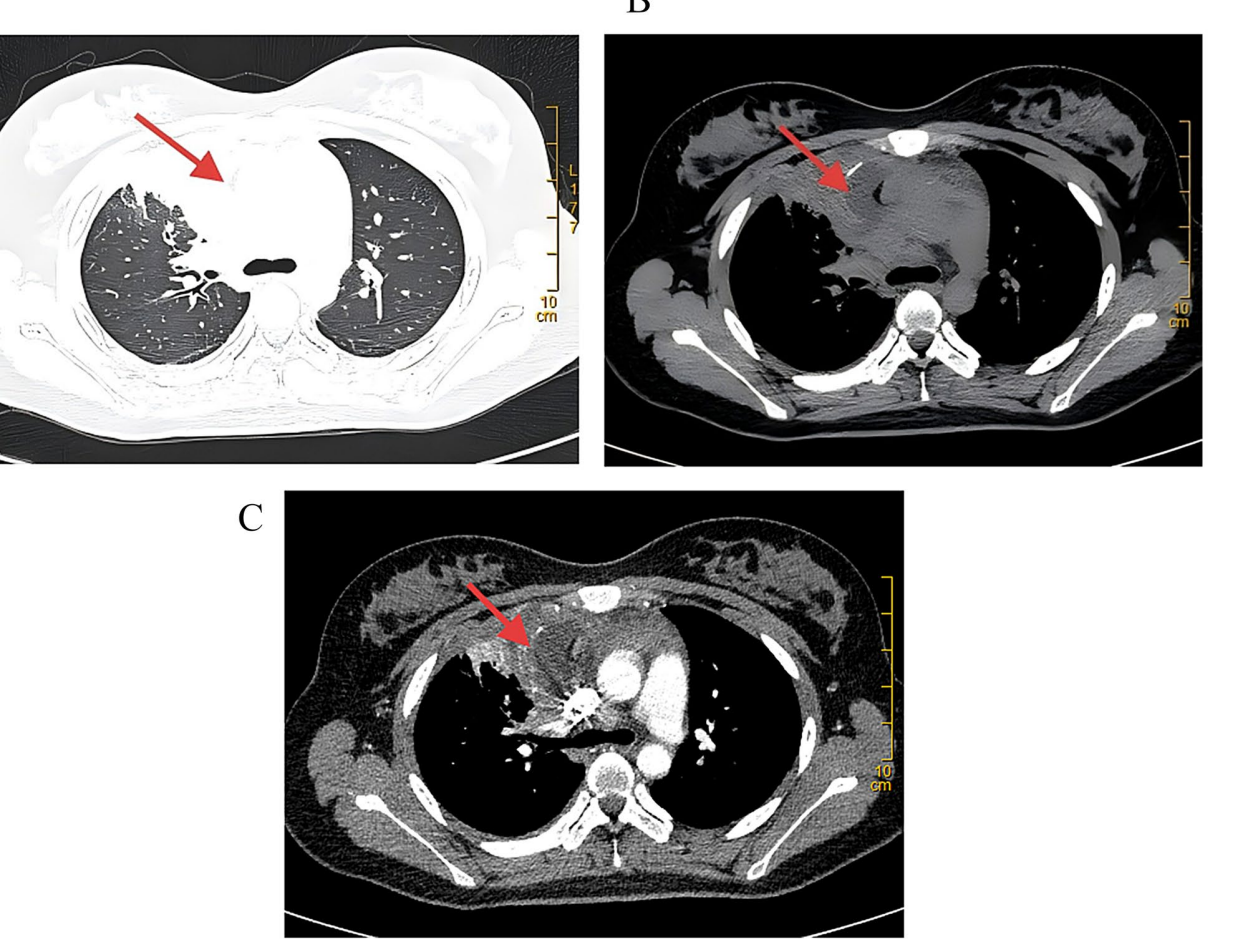
Chest computed tomography scan. **A**: In the upper lobe of the right lung, there are mass-like, nodular and patchy high-density shadows showing pulmonary involvement. **B**: A mixed density mass with patchy low density and gas shadows, along with air-fluid levels, is seen in the anterior mediastinum. **C**: After enhanced scanning, the mass showed heterogeneous enhancement, requiring histopathological assessment to distinguish infection, inflammation, or malignancy

The patient was subjected to high-resolution computed tomography (CT) analysis during breath holding at mid-inspiration using a GE Discovery CT750 HD 64. The scanning parameters were as follows: 120 kV, automatic tube current modulation (maximum 200 mA), slice thickness of 0.625 mm, and a field of view of 31.4 cm. Chest CT showed the presence of a patchy area of increased density in the right upper lobe of the lung. Some of them appeared as ground-glass changes, while others had soft tissue density. The boundary with the right mediastinal shadow was found to be unclear (Fig. 1A). A mixed-density mass measuring 7.3 × 4.8 × 7.1 cm was identified in the right anterior superior mediastinum (Fig. 1B). Post-contrast imaging demonstrated significant enhancement of the solid components within the mass (Fig. 1C).

Relevant laboratory tests during the patient’s hospitalization are as in Table 1. Blood investigations revealed a normal white cell count of 8.2 × 10^9^/L, normal serum immunoglobulin E and normal serum erythrocyte sedimentation rate. Pertinent tumor markers were within the normal range. The high-throughput detection of pathogenic microorganisms in deep sputum was negative. All tests for tuberculosis were negative, including acid‑fast bacillus smear and culture, purified protein derivative skin test, and interferon‑γ release assay.

**Table 1:** Laboratory parameters of the patient

Category	Test / Marker	Results	Units	Range/ Normal	Detection Method
Complete Blood Count	WBC Count	8.2	×10^9^/L	3.5–9.5	Flow Cytometry
	Neutrophils	47.8%	%	40–75	Flow Cytometry
	Eosinophils	2.3%	%	0.4-8	Flow Cytometry
Serum Tests	IgE	134	IU/ml	0−100	Nephelometry
	ESR	16	mm/h	0–20	Optical Method
Tumor Markers	AFP	2.6	µg/L	0–25	Electrochemiluminescence Assay
	NSE	12.52	µg/L	0-18.3	Electrochemiluminescence Assay
	CEA	1.4	ng/ml	<4.7	Electrochemiluminescence Assay
Microbiological Tests	High-throughput pathogen detection	Negative	--	Negative	mNGS
Tests for Tuberculosis	Sputum smear	Negative	--	Negative	Direct Smear Microscopy
	Sputum culture	Negative	--	Negative	Culture Method
	PPD test	Negative	--	Negative	PPD Skin Test
	IGRA	Negative	--	Negative	ELISpot

WBC, White Blood Cell Count; IgE, immunoglobulin E; ESR, Erythrocyte Sedimentation Rate; AFP, Alpha-fetoprotein; NSE, Neuron-specific enolase; CEA, Carcinoembryonic antigen; PPD, Purified protein derivative; IGRA, Interferon-γ release assay; mNGS, Metagenomic Next-Generation Sequencing; ELISpot, Enzyme-Linked Immunospot Assay

Based on imaging findings suggesting a mediastinal mass with adjacent infiltration, the patient was initially diagnosed with pneumonia complicated by mediastinitis. A 2-week course of antibiotic therapy was administered accordingly. The patient showed no clinical improvement and the laboratory test results were negative. Therefore, thoracoscopic surgery was performed to obtain a definitive histopathological diagnosis.

**Fig. 2:**
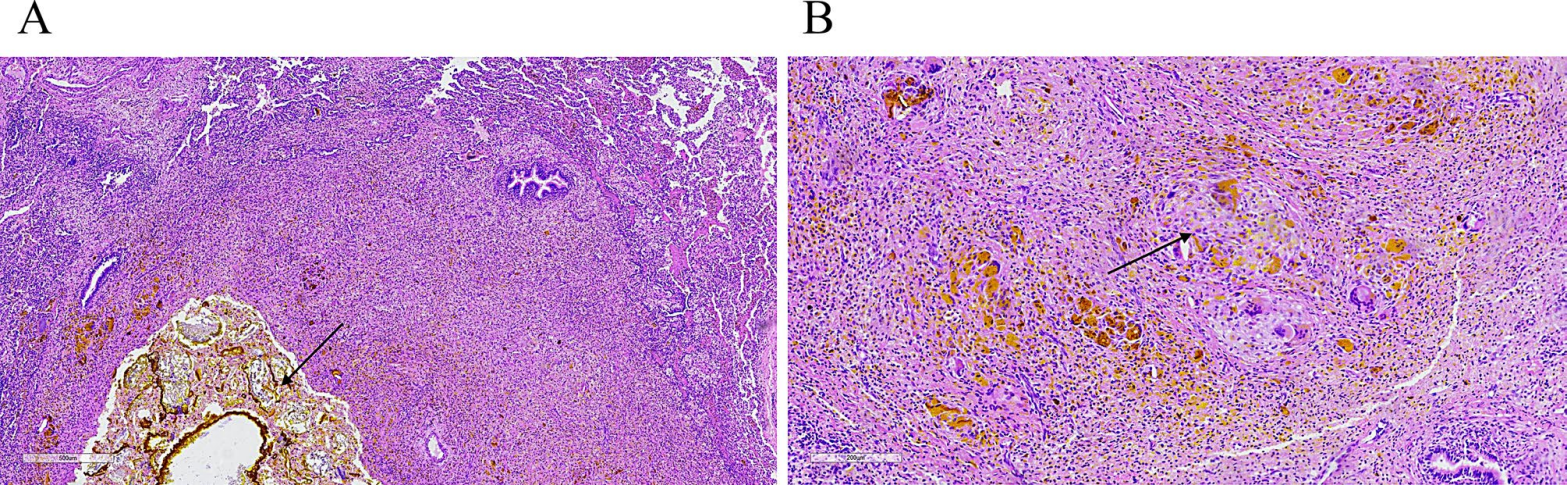
Pathological examination results of the sample. **A**: H&E staining (×40) revealed parasitic cystic structures containing necrotic debris, degenerated parasites, and neutrophilic infiltration, findings consistent with a parasitic infection. **B**: H&E-stained sections (×100) show a foreign-body granulomatous reaction in the surrounding tissue, a typical host response to chronic parasitic infection

The patient was placed in the left lateral decubitus position. A small incision was made at the fifth intercostal space along the right midaxillary line. The thoracic cavity was accessed through layer-by-layer dissection, followed by the insertion of a Trocar to establish a CO₂ pneumothorax. A thoracoscope was then introduced through the Trocar. Intraoperative findings revealed an irregular mass in the right anterior superior mediastinum. The mass was adjacent to the anterior segment of the right upper lobe and the right hilum. Its base adjoined the superior vena cava and bilateral brachiocephalic veins. Diffuse atelectasis was present in the left upper lobe anterior segment. Following meticulous dissection, the mass and adherent right upper lobe tissue were completely resected. Histopathological examination of the resected specimen revealed multiple parasitic cysts within the lung parenchyma, visceral pleura, and mediastinal mass tissue. The cysts contained necrotic debris, degenerated parasite bodies, and neutrophilic infiltration (Fig. 2A). Serofibrinous inflammation with adhesions of granulation and fibrous tissue was observed in the pleural and mediastinal specimens (Fig. 2B). The unexpected finding of parasitic structures, together with the histological morphology, raised strong suspicion of a parasitic infection, most notably paragonimiasis. Upon this preliminary diagnosis, further history-taking revealed that the patient habitually consumed undercooked crayfish and freshwater crabs. No evidence of ectopic infection was found on clinical examination or imaging. Repeated stool and sputum examinations using image analysis technology failed to detect parasite eggs. Furthermore, serological tests for antibodies against other parasites, including *Cysticercus*, *Sparganum*, *Plasmodium*, and *Clonorchis sinensis*, were performed using enzyme-linked immunosorbent assay (ELISA), and all results were negative.

The patient was prescribed an individualized course of praziquantel (25 mg/kg, administered twice daily for 15 days) based on the clinical and imaging findings. At a 1-year follow-up, the patient reported complete resolution of the respiratory symptoms. Although follow-up imaging was advised, the family of the patient declined further investigation as the child remained entirely asymptomatic. This individualized therapeutic strategy was formulated based on the patient’s specific clinical symptoms and imaging characteristics. Furthermore, this regimen was advised by experts at the Shandong Institute of Parasitic Diseases, China.

## Discussion and conclusions

*Paragonimus* species are foodborne trematodes that represent a re-emerging public health concern [10]. Paragonimiasis typically manifests with nonspecific respiratory symptoms such as cough, chest pain, and hemoptysis, and can even be asymptomatic [11]. Consequently, diagnosis is often challenging. The present case was notable for an unusual presentation: a mediastinal mass on imaging, accompanied by chronic cough.

Mediastinal masses in children could arise from congenital anomalies, benign or malignant neoplasms, or infectious processes [12, 13]. The differential diagnosis is clinically challenging due to the wide spectrum of possible etiologies. In the present case, several key conditions were carefully considered and excluded. Firstly, lymphoma, the most common mediastinal malignancy in this age group, was regarded as highly unlikely. Although lymphoma typically appears on CT as a large, bulky, homogeneous prevascular mass, the radiographic features of the lesion in our patient were distinctly atypical for this diagnosis. Secondly, germ cell tumors, a common etiology of anterior mediastinal masses in children, were ruled out. The lesion lacked characteristic imaging features of a teratoma, such as internal fat, fluid, calcification, or a fat-fluid level. Moreover, serum tumor markers provided no support for a malignant germ cell origin. Finally, pulmonary tuberculosis, which could manifest with nonspecific symptoms and mediastinal lymphadenopathy, was excluded based on the absence of exposure history, negative tuberculin skin test and interferon-gamma release assay results, and lack of typical radiographic findings. Thus, through combined radiographic, serologic, and clinical evaluation, these common diagnostic possibilities were reasonably excluded.

Pulmonary paragonimiasis is typically characterized on chest CT by findings such as tunnel signs, patchy infiltrates, nodules, calcifications, pleural effusion, or cavitation, and is often accompanied by eosinophilia [14]. In the present case, the absence of these characteristic deviated from the classic diagnostic profile. This absence aligns with several individual cases of pediatric ectopic paragonimiasis have been previously described(Table 2). While those cases involved diverse sites such as the brain, liver, and subcutaneous tissues, they also highlight that extra-pulmonary disease often lacks the typical imaging hallmarks of pulmonary involvement. Moreover, the absence of eosinophilia has been noted in chronic or non-recent infections [6, 15, 16]. This discrepancy underscores a critical limitation in relying solely on canonical laboratory and radiographic clues. Given the diagnostic uncertainty, nonspecific imaging, and failure of empirical antibiotics, thoracoscopic intervention was pursued due to the mass’s size and location.

**Table 2:** Overview of 6 patients with ectopic paragonimiasis

Author	Age(year)	Sex	Organ Involved	Presentation	Treatment	Outcome
Wang et al.[4]	7	Girl	Intraspinal	progressive weakness and paresthesias of both legs	Surgical excision + Praziquantel	Patient showed improvement clinically
Liu et al.[5]	11	Girl	Eyelid	severe right upper eyelid swelling and ptosis	Surgical excision + Praziquantel	mild eyelid scarring but no ocular or systemic dysfunction
Wang et al.[6]	10	Boy	Pericarditis	acute vomiting with fever and chills	Surgical excision + Praziquantel	stable cardiac function and no recurrence of pericardial effusion
Gong et al.[7]	1.3	Girl	Liver	persistent mild fever and hepatomegaly	Praziquantel	Patient showed improvement clinically
Ah-Rum et al.[8]	9	Girl	Abdominal Muscle	abdominal pain with anorexia	Praziquantel + Pleural effusion drainage	Patient showed improvement clinically
Singh et al. [9]	8	Boy	Brain	cough, fever, headache, and inability to move right arm	Praziquantel	Patient showed improvement clinically

**Fig. 3:**
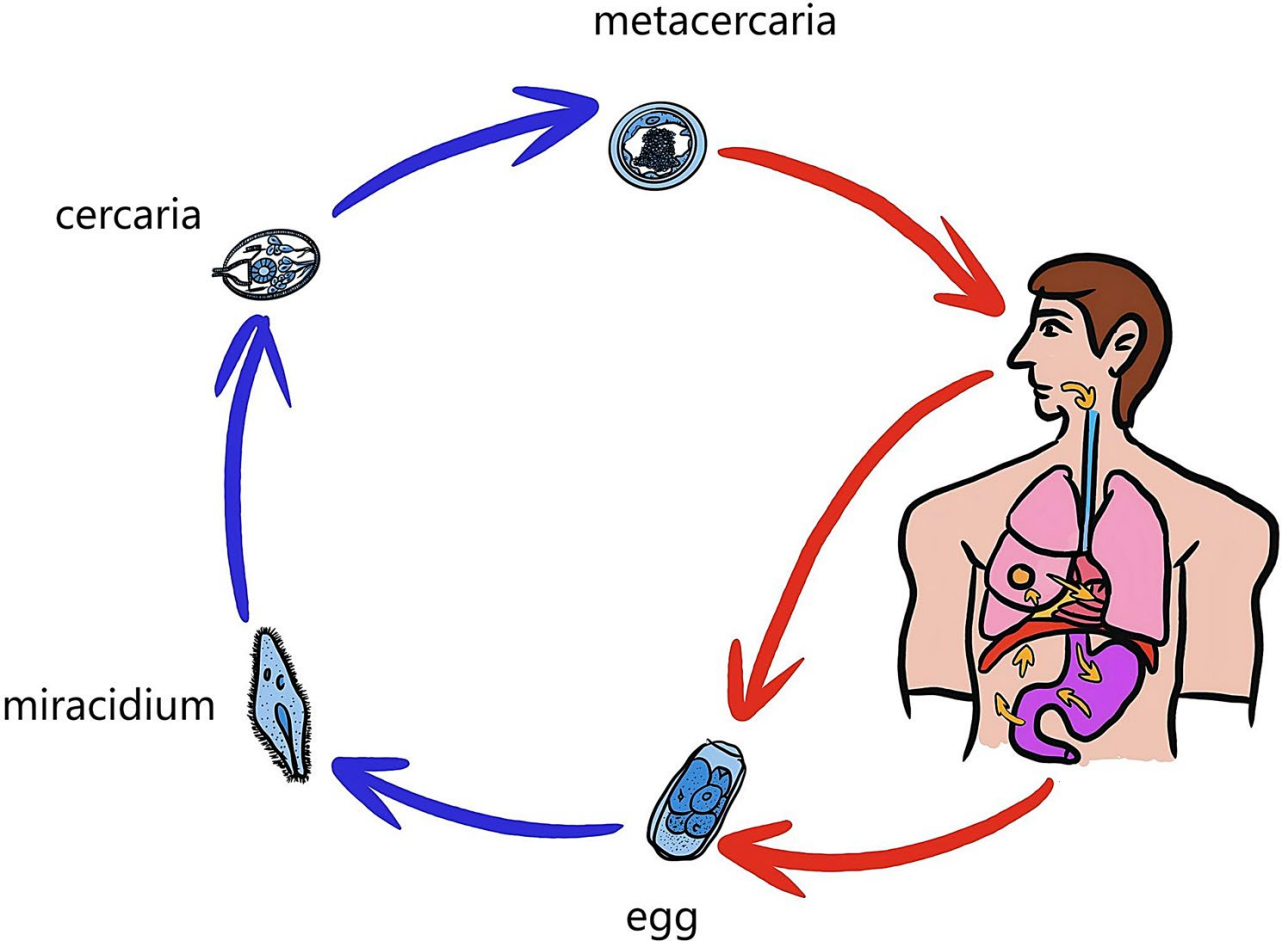
Life cycle of *Paragonimus*. *Paragonimus* eggs develop into metacercariae via two intermediate hosts. After human ingestion, metacercariae penetrate the intestinal wall and migrate as juvenile worms through the abdomen. They then cross the diaphragm into the thorax, reaching the lungs and mediastinum

Understanding the classification and life cycle of paragonimus is key to interpreting such atypical presentations (Fig. 3). Based on the final site of parasitic localization, paragonimiasis is classified into two forms: pulmonary and extra-pulmonary [17]. After ingestion, the larvae penetrate the intestinal wall, enter the peritoneal cavity, migrate through the diaphragm into the pleural space, and finally reach the lungs via the pleura. Aberrant migration can result in ectopic maturation in organs such as the liver, brain, or subcutaneous tissue [4–9].

Etiological confirmation remains the diagnostic gold standard. Appropriate specimens, including sputum, stool, or biopsy/surgical tissue, could reveal adult worms or eggs of *Paragonimus*, and then provide a definitive diagnosis [18]. The standard treatment regimen is praziquantel 25 mg/kg three times daily for 2–3 days. While this achieves satisfactory efficacy in most patients, treatment failure after the first course has been reported, sometimes requiring an extended or repeated regimen in severe or refractory cases [8, 19].

In conclusion, this case report demonstrates a rare instance of paragonimiasis presenting as a mediastinal mass. It therefore serves as an important reminder for pediatricians to maintain a high index of suspicion for this parasitic disease, even in the absence of classic clues such as eosinophilia or typical imaging findings. This experience suggests that in the evaluation of a pediatric patient with a mediastinal mass and chronic cough—particularly in an endemic region, with a history of consuming raw or undercooked freshwater crustaceans, and when symptoms are refractory to conventional therapy—paragonimiasis should be considered in the differential diagnosis. When the diagnosis remains elusive and empirical treatment is ineffective, as in the patient in the present case, timely histopathological evaluation through biopsy or surgical resection is crucial to avoid unnecessary diagnostic delay. Finally, we recommend avoiding the consumption of untreated water and ensuring that freshwater crustaceans are thoroughly cooked before consumption in endemic regions.

## Data Availability

The datasets generated and analyzed during the present study are available from the corresponding author on reasonable request.

## Abbreviations

CT: Computed tomography
ELISA: Enzyme-linked immunosorbent assay
